# Unresolved roles of Aux/IAA proteins in auxin responses

**DOI:** 10.1111/ppl.70221

**Published:** 2025-04-23

**Authors:** Monika Kubalová, Martina Schmidtová, Matyáš Fendrych

**Affiliations:** ^1^ Institute of Experimental Botany of the Czech Academy of Sciences Prague Czech Republic; ^2^ Department of Experimental Plant Biology Charles University Prague Czech Republic

## Abstract

Aux/IAA proteins are well‐known as key components of the nuclear auxin signaling pathway, repressing gene transcription when present and enabling gene activation upon their degradation. In this review, we explore the additional roles of Aux/IAA proteins in the known auxin perception pathways–the TIR1/AFBs nuclear as well as in the emerging cytoplasmic and apoplastic pathways. We summarize recent advances in understanding the regulation of Aux/IAA protein stability at the post‐translational level, a critical factor in auxin‐regulated transcriptional output. We further highlight the roles of auxin‐nondegradable non‐canonical Aux/IAAs in auxin‐mediated transcription and their involvement in apoplastic auxin signalling. Additionally, we discuss the importance of Aux/IAAs for the adenylate cyclase activity of TIR1/AFB receptors and speculate on their involvement in the cytoplasmic auxin pathway. Using Arabidopsis root as a model, this work underscores the central role of Aux/IAA proteins in mediating auxin‐driven developmental processes and environmental responses. Key questions for future research are proposed to further unravel the dynamic roles of Aux/IAAs in auxin signaling networks.

## 1 INTRODUCTION

AUXIN/INDOLE‐3‐ACETIC ACID (Aux/IAA) proteins are small, short‐lived proteins initially identified as early auxin‐responsive genes due to their strong upregulation following auxin application (Abel et al. 1994). In *Arabidopsis thaliana*, the Aux/IAA gene family comprises 29 members (Overvoorde et al. 2005). The structure of canonical Aux/IAA proteins includes three conserved domains, each contributing to distinct molecular interactions essential for their role in auxin signaling. Domain I recruits the TOPLESS (TPL) co‐repressor, which interacts with histone deacetylases and histone acetyltransferases to mediate transcriptional repression. Domain II contains a highly conserved “GWPPV” degron motif (Table 1) essential for auxin‐dependent binding to auxin receptors TRANSPORT INHIBITOR RESPONSE1 (TIR1)/AUXIN‐SIGNALLING F‐BOX (AFB) proteins. PHOX AND BEM1 (PB1) domain facilitates protein–protein interactions, enabling Aux/IAA proteins to oligomerize and interact with AUXIN RESPONSE FACTORS (ARFs) (Figueiredo and Strader 2022).

**TABLE 1:** Expression of Arabidopsis Aux/IAA genes in Arabidopsis root and their change after 20 min of IAA treatment (Kubalová et al. 2024) and 180 min induction of MPΔ (activation of auxin nuclear pathway) or AXR3‐1 (repression of auxin nuclear pathway) (Kubalová et al. 2025) TPM = transcripts per million. Statistically significant Fold change treatment/mock <0.5 = DOWN and >1.5 = UP.

gene_ID	symbols	degron	TPM in root	IAA 20 min	MPΔ 180 min	AXR3‐1 180 min
AT4G14560	IAA1, AXR5	GWPPV	**10.00**	UP	UP	DOWN
AT3G23030	IAA2	GWPPV	**42.93**	UP	UP	
AT1G04240	SHY2, IAA3	GWPPV	**24.18**	UP	UP	
AT5G43700	IAA4	GWPPV	**46.61**	UP	UP	
AT1G15580	IAA5	GWPPV	**0.22**	UP	UP	DOWN
AT1G52830	IAA6, SHY1	GWPPV	**1.11**	UP	UP	DOWN
AT3G23050	IAA7, AXR2	GWPPV	**69.94**			
AT2G22670	IAA8	GWPPI	**91.79**			
AT5G65670	IAA9	GWPPV	**323.91**			
AT1G04100	IAA10	GWPPL	**6.71**	UP		DOWN
AT4G28640	IAA11	GWPPI	**10.76**	UP	UP	
AT1G04550	IAA12, BDL	GWPPI	**21.39**		UP	
AT2G33310	IAA13	GWPPI	**54.30**	UP		
AT4G14550	IAA14, SLR	GWPPV	**32.55**			
AT1G80390	IAA15	GWPPV	**0.03**			
AT3G04730	IAA16	GWPPV	**293.80**		UP	DOWN
AT1G04250	AXR3, IAA17	GWPPV	**152.19**			
AT1G51950	IAA18	GWPPV	**16.00**			
AT3G15540	IAA19, MSG2	GWPPV	**11.93**	UP	UP	
AT2G46990	IAA20	‐ ‐ ‐ ‐ ‐	**1.10**			
AT3G16500	PAP1, IAA26	GWPPV	**23.72**			
AT4G29080	PAP2, IAA27	GWPPI	**19.86**	UP		
AT5G25890	IAA28, IAR2	GWPPV	**241.89**			
AT4G32280	IAA29	GWPPV	**0.74**			
AT3G62100	IAA30	‐ ‐ ‐ ‐ ‐	**1.34**		UP	UP
AT3G17600	IAA31	DWPPI	**2.31**		UP	DOWN
AT2G01200	IAA32, MEE10	‐ ‐ ‐ ‐ ‐	**0.17**		UP	
AT5G57420	IAA33	‐ ‐ ‐ ‐ ‐	**4.56**			
AT1G15050	IAA34	‐ ‐ ‐ ‐ ‐	**0.35**	UP		

The role of Aux/IAAs in the TIR1/AFBs‐dependent nuclear auxin pathway has been clarified: in the absence of auxin, Aux/IAA proteins bind ARFs through PB1 domain, repressing ARF‐mediated transcription via recruitment of TPL by Domain I. When auxin levels increase, Aux/IAA proteins are recognized by TIR1/AFB receptors through the degron motif in Domain II, leading to their ubiquitination by the SKP‐CULLIN‐F‐box (SCF)^TIR1/AFB^ complex and subsequent degradation via the 26S proteasome. This degradation process lifts the repression on ARFs, allowing for auxin‐responsive gene expression regulation (Leyser 2018). Additionally, Arabidopsis has six non‐canonical Aux/IAA proteins that lack typical Domains I and II (Overvoorde et al. 2005), precluding them from participating directly in the nuclear auxin pathway.

The transcriptional response to auxin, however, cannot fully account for all observed auxin responses. Studies on Arabidopsis roots have revealed that auxin's effects are not limited to transcriptional reprogramming but also include rapid, non‐transcriptional responses (Dubey et al. 2021); auxin is also perceived by membrane‐localized receptors of the TRANSMEMBRANE KINASE (TMK) family and, in hypocotyl tissues, the auxin signal is relayed to non‐canonical Aux/IAA proteins (Cao et al. 2019). Finally, there are numerous auxin‐independent inputs regulating the stability of Aux/IAA proteins (Luo et al. 2018).

This review summarizes recent discoveries and new insights regarding the dynamics and role of Aux/IAA proteins in auxin‐mediated transcription and speculates on their roles in non‐transcriptional auxin signaling. Additionally, it presents an overview of auxin's impact on Arabidopsis root development and growth responses, focusing on the involvement of Aux/IAA proteins. We aim to identify critical questions that remain in the study of Aux/IAA functions within auxin‐mediated responses to achieve a more comprehensive understanding of their roles in plant development.

## 2 THE MULTIFACETED REGULATION OF AUX/IAA PROTEIN LEVELS FINE‐TUNES AUXIN SIGNALING

**FIGURE 1:**
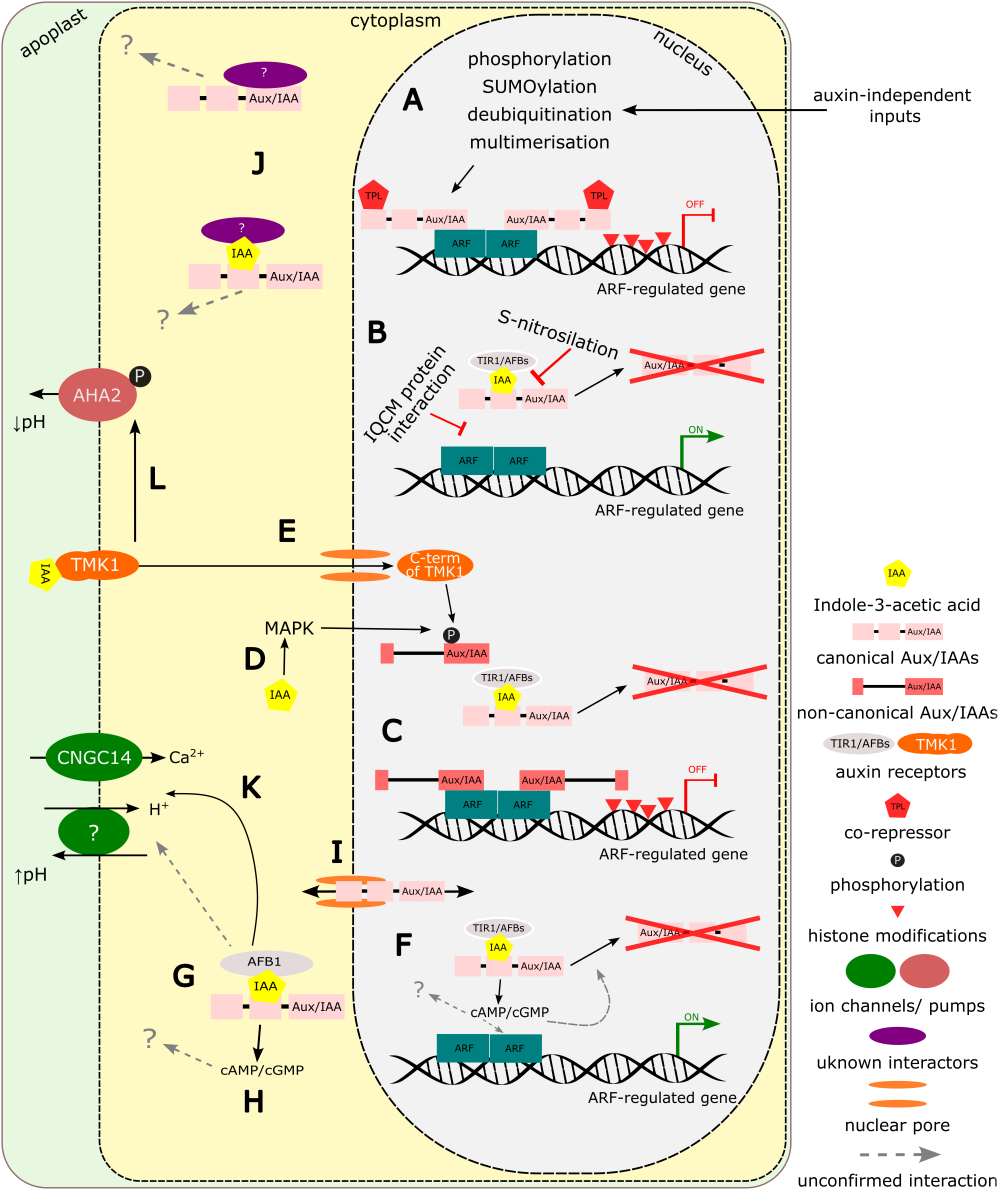
**Aux/IAA proteins in focus: their regulation and contribution to auxin signaling pathways. (A)** Canonical Aux/IAA (light pink) stability is key to regulating nuclear ARF‐dependent transcription. Low auxin levels and post‐translational modifications increase Aux/IAA protein stability, enhancing their repressive effect on transcription. **(B)** In contrast, high auxin levels facilitate canonical Aux/IAAs' interaction with TIR1/AFB receptors, leading to their degradation, though this binding can be reduced by S‐nitrosylation, while other protein interactions can block Aux/IAAs binding to ARFs, promoting ARF‐regulated transcription. **(C)** Non‐canonical Aux/IAA proteins (dark pink) regulate transcription by competing with and replacing canonical Aux/IAAs for ARF binding sites. They do not interact with TIR1/AFBs, avoiding degradation, but are post‐translationally stabilized in the presence of auxin. This stabilization occurs via **(D)** MAP kinase activation or **(E)** TMK1 receptor sensing auxin in the apoplast, triggering C‐terminal cleavage, nuclear translocation, and phosphorylation‐induced stabilization of non‐canonical Aux/IAAs. **(F)** The exact role of TIR1/AFB‐Aux/IAA co‐receptor‐mediated cyclic nucleotides production remains unclear. **(G‐I, K, L)** Cytoplasmic Aux/IAAs **(I)** could act as co‐receptors in the AFB1‐dependent cytoplasmic auxin pathway **(G)**, including possible cyclic nucleotide production **(H)**, which could affect ion transport **(K)** and counteract root acidification caused by TMK1 signaling **(L)**. **(J)** Additionally, Aux/IAA proteins relocated to the cytoplasm may interact with unknown partners in both auxin‐dependent and independent ways, influencing yet‐uncharacterized downstream processes.

The transcriptional regulation of auxin‐responsive genes relies on the degradation of Aux/IAA proteins (Leyser 2018), positioning them as central components of the nuclear auxin pathway. Therefore, their expression level and protein stability represent a critical factor in regulating the extent of the transcriptional response. Initially, the level of Aux/IAAs is determined by their expression regulation. Aux/IAAs were characterised as auxin‐inducible genes (Abel et al. 1994); yet, their expression is auxin‐independent to a large extent (Table 1) (Luo et al. 2018). Canonical Aux/IAA proteins are degraded in an auxin‐dependent manner; however, their stability is regulated by additional, auxin‐independent inputs (Figure 1A,B). Zhang et al. (2023) proposed that IAA17/AXR3 protein is stabilized through SUMOylation by the SUMO E3 ligase METHYL METHANESULFONATE‐SENSITIVE 21 (MMS21). Plants lacking MMS21 showed decreased levels of IAA17/AXR3, and SUMOylation was required for the root growth inhibitory effect caused by *IAA17/AXR3* overexpression. Exerting a similar effect on Aux/IAA protein stability, yet achieved by a different mechanism, UBIQUITIN‐SPECIFIC PROTEASE14/DA3 stabilizes IAA3/SHY2 through deubiquitination, coordinating auxin signaling in the pericycle and endodermis to control lateral root initiation (Peng et al. 2023). Heat‐responsive MITOGEN‐ACTIVATED PROTEIN KINASES (MAPKs) phosphorylate and stabilize IAA8 by decreasing its polyubiquitination; this leads to inhibition of flower development (Kim et al. 2024). Similarly, phosphorylation of IAA15 by MAPKs is necessary for the suppression of root development under drought stress in Arabidopsis (Kim et al. 2022). Furthermore, the S‐nitrosylation of IAA17/AXR3 inhibits its interaction with TIR1, negatively regulating auxin signaling by reducing the degradation of IAA17/AXR3 (Jing et al. 2023). On the other hand, CALMODULIN IQ‐MOTIF CONTAINING PROTEIN (IQCM) physically interacts specifically with IAA19 in a Ca^2+^‐dependent manner, weakening the interaction between IAA19 and ARF, allowing the activation of ARF‐dependent genes involved in callus formation and lateral root development (Zhang et al. 2022). In summary, several recent reports show that various inputs, including environmental factors, influence the regulation of Aux/IAA‐ARF‐dependent gene transcription by modulating the stability of Aux/IAA proteins, either reducing (Figure 1A) or enhancing (Figure 1B) their degradation rates.

Regulation of Aux/IAA levels thus represents a potent approach to modulate auxin signaling output. In fact, differing Aux/IAA levels can result in an opposite cellular response. Cho et al. (2024) suggested a model in which competition between Aux/IAA proteins with different TPL‐binding affinities creates a bimodal transcriptional switch: during root hair growth, IAA3/SHY2 acts both as an activator and repressor, depending on its concentration. In contrast, IAA7/AXR2 and IAA17/AXR3 consistently act as repressors of root hair growth. Interestingly, domain I determines whether an Aux/IAA protein will behave as a bimodal regulator (like IAA3/SHY2) or a consistent repressor (like IAA7/AXR2 or IAA17/AXR3), with consistent repressors showing stronger binding to TPL than those capable of switching functions.

Similarly, root cell elongation is highly dependent on the concentration of Aux/IAA proteins (Kubalová et al. 2024). Induction of stabilized, auxin non‐degradable *IAA17/AXR3 – AXR3‐1* led to loss of auxin sensitivity and boosted root cell elongation during the initial hours of accumulation. With the increasing level of AXR3‐1, the growth rate was inhibited, consistent with the described effect of stable AXR3‐1 overexpression (Knox et al. 2003).

Recent work proposed a model where Aux/IAAs capacity to form multimers plays a more critical role in xerobranching (a temporary suppression of root branching when water is limiting) than their protein levels. In this model, dynamic alterations in the cellular level of reactive oxygen species are detected by IAA3/SHY2. This detection triggers redox‐dependent multimerization of IAA3/SHY2, resulting in the formation of higher‐order multimers at specific target sites. These multimers are recruited through interactions with ARFs, such as ARF7. The nucleation of IAA3/SHY2 higher‐order multimers at these target sites enhances the surface for binding with a greater number of TPL co‐repressor molecules, resulting in more robust repression. However, the ability of these proteins to multimerize has not been observed in all tested Aux/IAA proteins (Roy et al. 2024). Therefore, multimerization seems to be another mechanism through which auxin fine‐tunes its Aux/IAA‐mediated responses.

Post‐translational modification of Aux/IAAs, interactions with other proteins or with themselves contribute to the level of Aux/IAAs, which is crucial for cellular responses. Interestingly, these regulatory mechanisms are not uniform across all Aux/IAAs despite their conserved sequences. On the contrary, these mechanisms appear to exhibit high selectivity for specific Aux/IAAs. Investigating the factors underlying the specificity of these modifications to different Aux/IAAs could provide valuable insights. Level of Aux/IAAs, together with varying affinities between different Aux/IAAs and TIR1/AFB co‐receptor pairs (Winkler et al. 2017; Calderón Villalobos et al. 2012), as well as ARF–Aux/IAA and Aux/IAA–Aux/IAA dimers (Farcot et al. 2015; Krogan and Berleth 2015; Lakehal et al. 2019), may help regulate the amplitude and speed of the auxin transcriptional response. Aux/IAAs specific expression and regulation may explain how a single auxin signal can trigger a variety of different responses.

## 3 NONCANONICAL AUX/IAAS INTERFERE WITH AUXIN‐REGULATED TRANSCRIPTION

Auxin controls both the degradation and expression of canonical Aux/IAAs (Abel et al. 1994). Six of the Arabidopsis Aux/IAA proteins (IAA20, IAA30, IAA31, IAA32, IAA33, and IAA34) differ from the rest of the group in that they lack the auxin‐interacting domain (Table 1), making them stable even in the presence of auxin. However, they are still involved in the regulation of auxin signaling (Figure 1C). The role and possible mechanism of action of these non‐canonical Aux/IAAs are discussed in the following part.

Lv demonstrated that non‐canonical IAA33 interacts with ARF10 and ARF16 to regulate root stem cell identity by competing with canonical IAA5 (Lv et al. 2020). *In vitro* pull‐down and three‐hybrid assays confirmed that IAA33 reduced the interaction between IAA5 and ARF10/ARF16, indicating competition between IAA33 and IAA5 for ARF binding. This is supported by *iaa33*, *iaa5*, and *IAA33/IAA5* overexpression lines having similar rate of root stem cell differentiation, with positive effects of knock‐out mutants and negative effects of overexpressing plants. The study also suggested that, while auxin does not regulate *IAA33* expression, it activates MAPK14, which stabilizes the IAA33 via phosphorylation (Figure 1D). How exactly auxin activates MAPKs has not been clarified. Similarly, the expression of another non‐canonical *IAA31* is not induced by auxin (Sato and Yamamoto 2008), while it remains untested whether IAA31 protein is stabilized by auxin‐induced activation of kinases.

Müller et al. (2016) demonstrated that the HD‐ZIP III transcription factors drive the expression of non‐canonical *IAA20* and *IAA30* in the root vasculature (Müller et al. 2016), thereby repressing ARF5/MONOPTEROS (MP) activity and ensuring a correct rate of protoxylem formation.

Two other non‐canonical Aux/IAA proteins, IAA32 and IAA34, intersect with the apoplastic auxin pathway (Cao et al. 2019). In this pathway, TMK receptors perceive extracellular auxin and relay the signal as phosphorylation of cytoplasmic or membrane proteins (Dai et al. 2013; Yu et al. 2023; Friml et al. 2022). In the apical hook of etiolated Arabidopsis hypocotyl, a site of high auxin accumulation (Žádníková et al. 2016), the C‐terminal domain of TMK1 is cleaved, moves to the nucleus where it phosphorylates IAA32 and IAA34 (Figure 1E). This phosphorylation stabilizes the IAA32 and IAA34 proteins, which then regulate the transcription of ARF2/ARF7 target genes, as demonstrated by transcriptome analysis of the *iaa32 iaa34* mutant. IAA32/34 activity contributes to the inhibition of cell elongation on the concave (inner) side of the apical hook, regulating the maintenance of its closure. At the same time, the inner side is the site of auxin signaling maximum, highlighted by the DR5 reporter maximum, which indicates high ARF activity (Žádníková et al. 2016). The two pathways, therefore, antagonize each other in the concave site of the apical hook. Interestingly, CYTOKININ INDUCED ROOT WAVING 1 (CKRW1)/WAVY GROWTH 3 (WAV3) E3 ubiquitin ligase mediates the ubiquitination and degradation of IAA32/34 in the convex (outer) side of the apical hook in an auxin‐independent manner (Wang et al. 2024). Further complicating the matter, the TMK pathway activates the plasma membrane proton AHA ATPases that, in turn, gate cell wall expansion by acidification (Walia et al. 2024; Lin et al. 2021); as a result, the hypocotyls of *tmk1/4* double mutant are dwarfed. The cleavage of the TMK receptor (required for the phosphorylation of non‐canonical Aux/IAAs) likely turns the TMK receptor inactive and, thus, unable the promotion of cell wall acidification and growth in the concave side of the hook. In the convex side, the receptor might activate AHAs in response to lower auxin levels. The various auxin signaling pathways intersect on the regulation of Aux/IAA stability in the apical hook; how exactly the auxin gradient steers the differential cleavage of TMK1 remains unclear. Peptidases from the DA1 family were, however, shown to regulate TMK1 cleavage in the convex side of the apical hook (Gu et al. 2022).

Non‐canonical Aux/IAA proteins represent an additional layer, freed from the regulation by TIR1/AFBs, that modulates ARF‐dependent transcription. In contrast to canonical Aux/IAA, protein levels of some of the non‐canonical Aux/IAA are positively regulated by the presence of auxin, increasing their inhibitory effect on transcription (Figure 1C). The decisive factor for non‐canonical Aux/IAA activity is thus the regulation of their expression, which is often tissue‐specific.

## 4 NEW LAYERS OF THE TIR1/AFB‐AUX/IAA SIGNALING

Recently, an active adenylate cyclase (AC) domain has been identified in TIR1/AFBs family auxin receptors (Qi et al. 2022). The AC activity is stimulated *in vitro* by the TIR1/AFBs–Aux/IAA co‐receptor assembly mediated by auxin (Figure 1F). Importantly, the AC activity itself is not required for the TIR1–Aux/IAA interaction, as two of the TIR1 mutant variants with abolished AC activity still could bind the IAA7 degron *in vitro* in an auxin‐dependent manner. Surprisingly, the TIR1 versions with compromised AC activity could only partially complement the ability of the roots to respond to auxin, as determined by both the long‐term root growth inhibition and the expression of auxin‐inducible genes. This means that cAMP (cyclic adenosine monophosphate) production by the TIR1/AFBs‐auxin‐Aux/IAA complex is required for a fully functional transcriptional response to auxin (Qi et al. 2022). These findings underscore the role of AC activity in events beyond the co‐receptor interaction, pointing towards its contribution to downstream signaling. What is this role? Is the cAMP signal needed for Aux/IAA degradation, for their dissociation from ARFs or for the activation of ARFs themselves (Figure 1F)? Do we need to rethink the model of Aux/IAA action in transcriptional response? Could their primary role be the production of the cAMP signal? A lot of questions remain to be resolved.

In Arabidopsis roots, the induction of expression of the dominant *IAA17/AXR3 ‐ AXR3‐1*, unable to assemble into the co‐receptor due to the mutation in the degron domain, triggers transcriptional changes that cause an uncontrolled cell elongation and loss of gravitropism (Knox et al. 2003; Kubalová et al. 2024). Analogously, the induction of a dominant version of ARF5/MP ‐ MPΔ (lacking the C‐terminal PB1 domain that is required for interaction with the Aux/IAA proteins) leads to an almost complete root growth arrest within 3 hours of MPΔ induction (Kubalová et al. 2024). This demonstrates that neither AXR3‐1 nor MPΔ seem to require the activity of TIR1/AFBs‐Aux/IAA co‐receptor for their activity (Krogan et al. 2012). This indicates that both the Aux/IAAs and ARFs can control transcription, without a need for the rise in cAMP levels, when overexpressed in roots. The role of the cAMP second messenger in the events downstream of the co‐receptor assembly thus remains enigmatic.

In their newest work, Chen et al. (2025) show that cAMP production is not required for Aux/IAA degradation, but rather necessary for the ability of ARFs to regulate gene transcription. Intriguingly, they showed that local cAMP production in the vicinity of the Aux/IAA–ARF complex induced ARF transcriptional activity.

It is intriguing to consider the temporal dynamics of the auxin signaling events. In a low‐auxin situation, the level of Aux/IAAs is high, and the level of cAMP production is low. During auxin concentration increase, cAMP production increases as Aux/IAAs‐TIR1/AFBs co‐receptor assembles; however, the concentration of Aux/IAA proteins declines, presumably leading to a lower cAMP production. Later, the negative transcriptional feedback increases the expression of Aux/IAAs (Abel et al. 1994), likely increasing the rate of cAMP production. It will be intriguing to analyze the dynamics and correlation of cAMP levels with the degradation rates of Aux/IAAs during auxin response on a cellular level using genetically encoded cyclic nucleotide sensors (Massengill et al. 2021) and auxin response sensors.

The instant production of cAMP upon perception of auxin by the TIR1/AFBs‐Aux/IAA complex is intriguing, as it could represent the missing link between the TIR1/AFBs and the ultra‐rapid auxin responses such as membrane depolarization, calcium influx into cells and root growth inhibition (Fendrych et al. 2018; Dubey et al. 2023; Serre et al. 2023; Shih et al. 2015; Serre et al. 2021). However, Qi et al. (2022) disproved this hypothesis by showing that the TIR1 with compromised AC activity inhibits root growth as rapidly as its wild‐type control and also triggers a comparable Ca^2+^ signature. There is, however, one remaining caveat. The ultrarapid responses of roots to auxin depend specifically on the AFB1 paralog, as discovered by Prigge (2020). AFB1 is required for the auxin‐induced membrane depolarization and CNGC14 (CYCLIC NUCLEOTIDE‐GATED CHANNEL)‐dependent Ca^2+^ influx (Shih et al. 2015; Serre et al. 2023; Dubey et al. 2023). The roots of *afb1* single loss of function mutants miss the rapid response to auxin completely, and TIR1 cannot replace the AFB1 function (Chen et al. 2023; Dubey et al. 2023). Therefore, the conclusions drawn from the TIR1 effects on ultra‐rapid responses should be expanded to the AFB1 paralogue (Figure 1G).

Bringing a new twist to the story, it appears that TIR1/AFBs harbor a guanylate cyclase (GC) domain located next to the AC domain. Similarly to the AC activity, the production of cGMP (cyclic guanosine monophosphate) is stimulated upon the TIR1/AFB‐auxin‐Aux/IAA co‐receptor complex formation (Figure 1H), as shown *in vitro* for the TIR1‐IAA7 or TIR1‐IAA17/AXR3 and for AFB1‐IAA7 co‐receptor. The GC activity itself is not needed for the co‐receptor formation *in vitro*, analogously for the situation of AC activity (Qi et al. 2023). Treatment with IAA (Indole‐3‐Acetic Acid) led to an increase in cAMP and cGMP content; while cAMP levels increased relatively slowly, the levels of cGMP increased within 1 minute of treatment. Interestingly, this rapid increase requires the presence of multiple TIR1/AFB receptors, as the increase was not detectable in the *tir1‐1 afb2‐1 afb3‐1* triple mutant and decreased in the *afb1* mutant. The genetics of the cGMP increase is thus not fully consistent with the genetic analysis of the ultra‐rapid root responses —which are dominated by the AFB1 receptor, while the other TIR1/AFB receptors are dispensable (Dubey et al. 2023). The authors (Qi et al. 2023) propose that the cGMP spike produced by the AFB1 activates the plasma membrane calcium channel CNGC14, thereby triggering the ultrarapid ion flux changes and root growth inhibition in Arabidopsis roots. The genetic confirmation of this hypothesis is still, however, missing.

These new fascinating findings bring an additional layer of complexity to the functioning of the TIR1/AFB‐Aux/IAA auxin co‐receptors and indicate that cAMP and possibly cGMP function as second messengers in auxin signaling. The moonlighting function (Jeffery 1999) of TIR1/AFB‐Aux/IAA co‐receptors prompts a re‐evaluation of the nuclear auxin pathway model as well as the functioning of the rapid, non‐transcriptional responses to auxin that resides in the cytoplasm.

## 5 SUBCELLULAR LOCALIZATION AND DYNAMICS OF AUX/IAA PROTEINS

The components of the nuclear auxin pathway, in particular the Aux/IAA proteins, would be expected to localize to the nucleus, where they interact with both the TIR1/AFBs and the ARFs (Leyser 2018). Supporting this, all components of the nuclear auxin pathway from Marchantia are localized in the nucleus (Das et al. 2024). While there are single copies for each component in this minimal system, it is likely that auxin response components in this species act only in the nucleus, which may reflect their ancestral role. However, a detailed analysis showed that some of the Arabidopsis TIR1/AFBs partition between the nucleus and cytoplasm (Prigge et al. 2020). Later, it was discovered that AFB1 functions in the cytoplasm, where it triggers ultra‐rapid responses to auxin (Dubey et al. 2023). Interestingly, TIR1/AFBs is not alone to be localized outside of the nucleus. Powers et al. (2019) showed that while two class‐A (activating) ARFs were nuclear in the root meristem, they formed cytoplasmic condensates in the differentiated cells of the upper root. Moreover, the cells with cytoplasmic condensates were less sensitive to auxin. The authors suggested that auxin sensitivity can be modified cell‐specifically by presumably reversible nucleo‐cytoplasmic partitioning of the responsive apparatus. Little is known about how ARFs change their localization, but it certainly provides an additional dynamic layer to the regulation of auxin signalling (Powers et al. 2019, see also Jing et al. 2022). Is the nucleus indeed the exclusive subcellular localization of Aux/IAAs?

Aux/IAAs, despite having two quite conserved nuclear localization sequences (Abel et al. 1994), also appear to be cytoplasmic in some cases. There are studies reporting cytoplasmic Aux/IAAs in maize (Ludwig et al. 2014), tomato (Audran‐Delalande et al. 2012) and Arabidopsis (Arase et al. 2012).

However, the cytoplasmic localization of Aux/IAAs is often observed in heterologous systems such as leaf mesophyll protoplasts or transient expression in tobacco leaves, which can differ from natural conditions. Zhang et al. (2019) described that IAA17/AXR3 was exclusively nuclear in the roots of stable transgenic overexpressing lines, while it appeared partially cytoplasmic when expressed transiently in both *Arabidopsis* and tobacco leaves. Intriguingly, a recent study showed a dynamic localization of two Aux/IAAs. In the elongation zone of Arabidopsis root, IAA12 and IAA19 were nuclear and cytoplasmic (Figure 1I), but they turned exclusively nuclear in abiotic stress conditions. This localization change is regulated by a component of the nuclear pore complex (Nam et al. 2023). This relocalization of Aux/IAAs could possibly affect the cell's sensitivity to auxin, analogously to the relocalization of ARFs. The cytoplasmic pool of Aux/IAAs is ‘invisible’ for the nuclear auxin pathway; however, at the same time, it represents a possible co‐receptor for the cytoplasmic AFB1 pathway (Figure 1G) and, as such, could stimulate the cyclic nucleotide production and thus steer ultra‐rapid auxin responses (Qi et al. 2023; Dubey et al. 2023). Additionally, cytoplasmic Aux/IAAs may interact with other yet‐to‐be‐discovered interactors in both auxin‐dependent and independent manner (Figure 1J).

## 6 ARABIDOPSIS ROOT AS A MODEL FOR DISSECTING AUX/IAA CONTRIBUTIONS TO AUXIN SIGNALING PATHWAYS

The *Arabidopsis thaliana* root represents an excellent model system to assess the role of Aux/IAA proteins in various auxin response pathways – the canonical nuclear auxin pathway, rapid AFB1‐mediated cytoplasmic auxin responses, the roles of non‐canonical Aux/IAAs, as well as the TMK‐dependent apoplastic auxin pathway. Gene expression regulation of many Aux/IAAs in Arabidopsis root is dynamically regulated by auxin (Kubalová et al. 2025; Kubalová et al. 2024) (Table 1).

The dominant version of IAA17/AXR3, AXR3‐1, which inhibits the nuclear auxin pathway, exhibits a dual effect on root cell elongation: initial accumulation promotes excessive cell elongation, whereas prolonged accumulation results in growth inhibition (Kubalová et al. 2024). Interestingly, different auxin‐dependent processes in roots, such as gravitropism, cell elongation, and root hair growth, show differing sensitivities to the level of AXR3‐1. Additionally, SHY2/IAA3 displays bimodal activity – promoting root hair growth at low doses and inhibiting it at higher concentrations (Cho et al. 2024). Aux/IAA protein levels regulate precisely auxin transcriptional outputs in roots.

Aux/IAA proteins may potentially act as co‐receptors of the cytoplasm‐localized AFB1 protein (Dubey et al. 2023); indeed, IAA12 and IAA19 have been observed in the cytoplasm of elongating root cells (Nam et al. 2023). Since AFB1 exhibits reduced interaction with CULLIN1 (CUL1), resulting in inefficient assembly into the SCF complex (Yu et al. 2015), interactions between Aux/IAAs and AFB1 are unlikely to lead to the ubiquitination and degradation of Aux/IAAs as seen in the transcriptional pathway. Instead, it is possible that, upon auxin binding, these Aux/IAAs stimulate AFB1's production of cyclic nucleotides (Figure 1G) (Qi et al. 2023). Interestingly, when the nuclear localization signal of the dominant AXR3‐1 was mutated, allowing it to localize to the cytoplasm, it exerted a negative effect on root cell elongation (Kubalová et al. 2024). At present, this effect lacks a simple explanation, as the mechanism of AFB1 pathway functioning remains unknown. However, it is evident that while the AFB1‐dependent signaling is crucial for regulating rapid root gravitropic responses (Serre et al. 2021), the nuclear auxin pathway continues to play a dominant role in long‐term gravitropic root responses (Kubalová et al. 2024).

Another battlefield where auxin response pathways counteract each other is the regulation of root surface pH. The AFB1‐dependent pathway drives root surface alkalinization (Figure 1K) (Serre et al. 2022). The nuclear auxin pathway, while promoting acidification in above‐ground organs (Fendrych, Leung, and Friml 2016), contributes to surface pH alkalinization in roots (Friml et al. 2022; Li et al. 2021). Manipulating auxin signaling using dominant AXR3‐1 and dominant ARF5/MPΔ leads to surface acidification and alkalinization, respectively (Kubalová et al. 2024). Conversely, the TMK1 pathway stimulates AHA H^+^ ATPases in response to auxin (Figure 1 L) (Li et al. 2021; Friml et al. 2022), acting in opposition to the previously mentioned pathways. The mechanisms of how these three pathways control surface pH, how this is connected to cell elongation, and the importance of Aux/IAA proteins remain unclear.

The large number of Aux/IAAs, their specific interactions with auxin receptors, their diverse regulation by auxin, and their subcellular localization —regulated at least in part by environmental conditions— confirm that these small proteins play a crucial role in the plant's varied responses to auxin. Do Aux/IAAs link all the auxin perception pathways —nuclear, cytoplasmic and apoplastic? How is precise regulation of canonical and non‐canonical Aux/IAAs achieved at time and tissue level? What is the biological function of cytoplasmic Aux/IAAs? Are Aux/IAAs involved in the rapid auxin response? If so, do they act as co‐receptors of AFB1 for auxin? Is cyclic nucleotide production induced by Aux/IAA‐AFB1 binding involved in the rapid auxin response? These and many other unanswered questions ensure that studying Aux/IAA proteins will remain an exciting field for a long time.

## AUTHOR CONTRIBUTIONS

Conceptualization: MF, MK; Writing – original draft, review & editing: MF, MK, MS; Visualization: MK.

## FUNDING INFORMATION

MF, MK and MS received support from the European Research Council (Grant No. 101125499). MK was supported by Charles University Grant Agency (Grant No. 337021). MF was supported by the project TowArds Next GENeration Crops, reg. no. CZ.02.01.01/00/22_008/0004581 of the ERDF Programme Johannes Amos Comenius.

## Data Availability

Non‐applicable.
